# 

**Published:** 1963-07

**Authors:** 


					/ OA Jnlw 1963
t^r'npp T n
'1 ?. . ? ^ t
v.-v; ^ ?.\ ^ 'I ..? | j '
_ ^ ? T? ; ? - !! I j i I
:' iHi' ! inn i..'i
INCORPORATING the medical journal of the south west
Vol 78 (iii) NO 289
?

				

